# Metal-Promoted Higher-Order Assembly of Disulfide-Stapled Helical Barrels

**DOI:** 10.3390/nano13192645

**Published:** 2023-09-26

**Authors:** Ashutosh Agrahari, Mark Lipton, Jean Chmielewski

**Affiliations:** Department of Chemistry, Purdue University, 560 Oval Drive, West Lafayette, IN 47907, USA; aagraha@purdue.edu

**Keywords:** helical barrel, amphiphilic peptide, higher order assembly, biomaterial, 3D matrix, cargo

## Abstract

Peptide-based helical barrels are a noteworthy building block for hierarchical assembly, with a hydrophobic cavity that can serve as a host for cargo. In this study, disulfide-stapled helical barrels were synthesized containing ligands for metal ions on the hydrophilic face of each amphiphilic peptide helix. The major product of the disulfide-stapling reaction was found to be composed of five amphiphilic peptides, thereby going from a 16-amino-acid peptide to a stapled 80-residue protein in one step. The structure of this pentamer, **5HB1**, was optimized in silico, indicating a significant hydrophobic cavity of ~6 Å within a helical barrel. Metal-ion-promoted assembly of the helical barrel building blocks generated higher order assemblies with a three-dimensional (3D) matrix morphology. The matrix was decorated with hydrophobic dyes and His-tagged proteins both before and after assembly, taking advantage of the hydrophobic pocket within the helical barrels and coordination sites within the metal ion-peptide framework. As such, this peptide-based biomaterial has potential for a number of biotechnology applications, including supplying small molecule and protein growth factors during cell and tissue growth within the matrix.

## 1. Introduction

**Figure 1:**
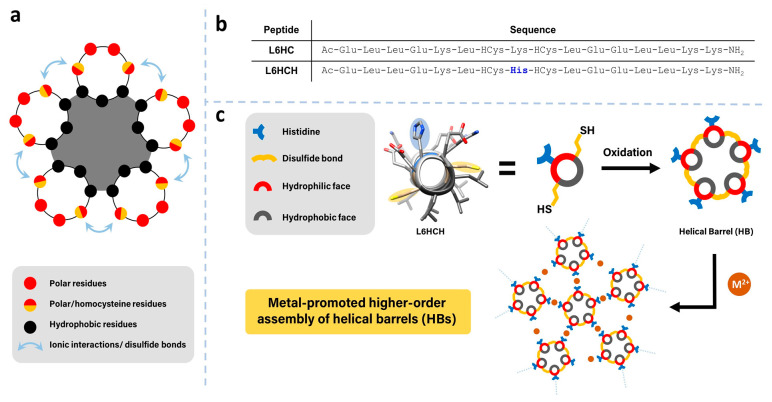
(**a**) The design of helical peptide interactions within helical barrels. (**b**) Sequences of **L6HC** and **L6HCH**. (**c**) Schematic representation of metal-promoted higher order assembly from a disulfide-stapled, helical barrel building block.

Peptides have been key elements for the development of biomaterials, with a rich landscape of secondary and super-secondary structures used as building blocks, including amphiphilic peptides, peptide amphiphiles, collagen mimetic peptides and coiled coils [1,2,3,4]. Coiled coils, for instance, contain two or more helices wrapped around each other via interhelical hydrophobic and complimentary charge interactions (Figure 1a). As the arc of the hydrophobic face of each helical peptide increases, larger oligomers result, leading to the formation of helical bundles and barrels from coiled coils involving four and five or more amphiphilic helices, respectively (Figure 1a). These designed helical barrels have been stabilized by interhelical ionic interactions between the coiled coils as described in the work of Woolfson, Conticello and Montclare [5,6,7,8,9,10,11,12,13,14,15]. Interhelical disulfide linkages have also been used to stabilize helical barrels starting with shorter coiled coil peptides as described by Chmielewski and coworkers [16]. 

An interesting property of helical barrels is that they exhibit a hydrophobic cavity that increases in size depending on the number of helices involved [5,6,7,9,10,15,16,17]. The hydrophobic cavity has been shown to have strong binding affinity for hydrophobic guests, including diphenylhexatriene, Prodan, curcumin, farnesylated and geranylgeranylated molecules and hydrophobic dyes [5,11,12,14,15,16,18]. The presence of the cavity within these barrels makes the oligomers excellent candidates for biomaterial applications [11,12,13,14,15,19,20]. For instance, Woolfson and Conticello have designed strategies using complimentary charge recognition and hydrophobic interactions at the termini for the higher order assembly of helical barrels into peptide-based nanotubes and fibers [11,15]. Montclare and coworkers have reported assembly of helical barrels containing an N-terminal His-tag into fibers, with changes to the morphology and hydrophobic cargo binding in a metal-ion-dependent fashion [12]. 

In this work, we disclose a strategy for metal-promoted higher-order assembly of helical barrels using the disulfide-linked barrel reported by Chmielewski and coworkers as a building block [16]. To facilitate metal-promoted higher-order assembly of the helical barrels, we have incorporated a His residue as a ligand for metal ions near the center of each helix of the disulfide crosslinked helical barrel. Using in silico molecular modeling, we have optimized the structure of the resultant helical barrels to evaluate the hydrophobic cavity within the barrels and have demonstrated hydrophobic dye binding. We demonstrate these helical barrels form a three-dimensional (3D) matrix in the presence of bivalent metal ions, which can be decorated with hydrophobic dyes through interactions with the barrel cavity and with a His-tagged green fluorescent protein through metal–ligand interactions. 

## 2. Materials and Methods

### 2.1. Materials

All N-Fmoc- and orthogonally sidechain-protected amino acids, including Fmoc-S-acetamidomethyl-L-homocysteine, and peptide synthesis reagents were acquired from Chem-Impex Inc. (Wood Dale, IL, USA) and ChemPep. Fisher Scientific (Wellington, FL, USA) provided all of the solvents used, and Sigma-Aldrich (St. Louis, MO, USA) provided the riisopropylsilane (TIPS). Alfa Aesar (Ward Hill, MA, USA) provided the diisopropylethylamine (DIEA) along with the metal salts used in the experiment described. Trifluoroacetic acid (TFA) was purchased from Acros Organics (Fair Lawn, NJ, USA). The Rink Amide resin was bought from Gyros Protein Technologies (Uppsala, Sweden). Acridine orange nonyl bromide was purchased from Thermo Fisher (Eugene, OR, USA). Dialysis cassettes (2000 Da MW cutoff) were purchased from Thermo Fisher. 

### 2.2. General Peptide Synthesis and Purification

**L6HCH** was prepared on a Symphony (Gyros Protein Technology) peptide synthesizer using standard Fmoc chemistry on Rink amide resin (0.45 mmol/g). In a typical synthesis, the resin was treated with Fmoc-Glu(OtBu) (4 eq.), 1-[bis(dimethylamino)methylene]-1H-1,2,3-triazolo[4,5-b]pyridinium-3-oxid hexafluorophosphate) (HATU, 4 eq.) and N,N-diisopropylethylamine (DIEA) (8 eq.) for 2.5 h in dimethylformamide (DMF). The resin was washed three times each with DMF, dichloromethane (DCM), methanol (MeOH), DCM, and DMF. The Fmoc-protecting group of the added Fmoc-Glu(OtBu) was removed with 25% piperidine in DMF for 30 min. The resulting resin was washed following the above mentioned solvent sequence before treatment with the next amino acid. These steps were iterated for the rest of the amino acids in the peptide sequence. After Fmoc deprotection of the final amino acid, the acetyl group was installed using 5% acetic anhydride in DMF (*v*/*v*) with 8.5% DIEA for 25 min. The resin was washed three times with DMF and DCM.

After drying the resin under reduced pressure, the peptide was cleaved from the resin using a TFA cocktail (95% TFA, 2.5% TIPS, 2.5% H_2_O) for 2.5 h. The resin was washed with the cleavage cocktail (1×) and DCM (2×). The solvent from the combined washes was removed in vacuo. Cold diethyl ether was used to precipitate the peptide from solution, and the solid was obtained by centrifugation and dried in vacuo. The crude solid containing **L6HCH** was dissolved in water and purified to homogeneity using reverse-phase high-performance liquid chromatography (RP-HPLC) with solvent A (CH_3_CN/0.1% TFA) and solvent B (H_2_O/0.1% TFA) as mobile phases on a Luna C18 semi-prep column over 60 min with an eluent gradient of 10% to 80% solvent A and a flow rate of 10 mL/min with detection at 214 nm. The resulting pure peptide was analyzed using matrix-assisted laser desorption ionization time-of-flight (MALDI-ToF) mass spectrometry (mass calculated: 2151.2, mass observed: 2153.0) (Figures S1–S3).

### 2.3. Interhelical Disulfide Bond Formation

In order to remove the acetamidomethyl (Acm) protecting group and form the disulfide crosslinks, a solution of **L6HCH** in water (0.5 mg/mL to 2 mg/mL) was treated with cyanogen iodide (100 eq.) [16,21]. The reaction mixture was wrapped in aluminum foil and stirred for 24 h, followed by 24 h dialysis with a dialysis cassette (2000 Da MW cutoff). The resulting mixture was separated using RP-HPLC using solvent A (CH_3_CN/0.1% TFA) and solvent B (H_2_O/0.1% TFA) as mobile phases on a Luna C18 semi-prep column over 60-min with an eluent gradient of 5% to 70% solvent A, and a flow rate of 10 mL/min with detection at 214 nm. The isolated products were characterized using MALDI-ToF mass spectrometry. The calculated and observed masses of isolated products are as follows: **4HB1:** mass calculated: 8028.4, mass observed: 8023.0; **4HB2:** mass calculated: 8028.4, mass observed: 8029.4; **4HB3:** mass calculated: 8028.4, mass observed: 8032.3; **5HB1:** mass calculated: 10,035.5, mass observed: 10,033.5 (Figure S5); **5HB2:** mass calculated: 10,035.5, mass observed: 10,036.3; **5HB3:** mass calculated: 10,035.5, mass observed: 10,034.2; **5HB4:** mass calculated: 10,035.5, mass observed: 10,034.1.

### 2.4. Circular Dichroism Spectroscopy

The circular dichroism (CD) was used to monitor the folding of the peptides using a JASCO J-810 CD spectropolarimeter (Jasco Inc.) (Easton, MD, USA) over three scans between 190 and 260 nm using solutions of peptides (25 μM) in phosphate buffer (20 mM, pH 7.2) at room temperature. The data pitch and bandwidth were set to 1 nm with the scan rate 100 nm/min and a 1 s digital integration time. Temperature scans were performed using the same setting with a 2 °C temperature interval from 4 to 90 °C with a temperature gradient of 2 °C/min and an equilibration time of 60 s for scans at each temperature. Helicity (%)was calculated from the mean residue ellipticity (MRE) at 222 nm using the following equation: % helix = 100([ϴ]_222_/(−39,500(1 − 2.57/n))), where n is the number of residues [22].

### 2.5. Molecular Modeling

The supramolecular structures of the helical barrels were prepared using the Maestro interface (Schrödinger, Inc.) (New York, NY, USA) and structural optimization was performed using the MacroModel molecular mechanics suite [23] The Amber94 force field [24] was employed with the GB/SA continuum solvent model [25] simulating an aqueous environment. Energetic minimization was performed using the truncated Newton method of Ponder and Richards [26]. This was followed by a 2 ns stochastic dynamics simulation [27] (300 K, 200 ps equilibration time) to further optimize the structure of the supermolecular complex.

### 2.6. Fluorescence Spectroscopy

Fluorescence measurement was used to monitor acridine orange nonyl bromide (AONB) binding to **5HB1** using a Tecan Infinite F Plex plate reader with a Coaster 96 flat bottom black polystyrene plate at room temperature. An excitation wavelength of 485 ± 20 nm and emission wavelength of 535 ± 25 nm was used with optimal gain setting and 20 μs integration time. The concentration of the aqueous solution of AONB was kept at 1 μM, while the concentration of **5HB1** in water was varied from 1 to 50 μM.

### 2.7. Metal-Promoted Assembly

To a microtube with water (27.5 μL) and 3-(N-morpholino)propanesulfonic acid (MOPS) buffer (12.5 μL, 200 mM, pH = 7.4), **5HB1** (5 μL, 5 mM) was added followed by addition of metal salts (2 eq. or 5 eq.) (ZnCl_2_ or CuCl_2_ or FeCl_2_) to promote higher-order assembly. The final volume of the mixture was 50 μL with final concentrations of 50 mM for MOPS buffer, 0.5 mM for **5HB1** and 1 or 2.5 mM for metal ion. The mixture was incubated at room temperature for 1 h followed by centrifugation at 5000× *g* for 3 min. The supernatant was removed, and the pellet was washed twice with water.

### 2.8. Scanning Electron Microscopy

An aliquot (3 μL) of the washed assembly of **5HB1** was placed on a glass side attached with copper tape to a metal stub, followed by air drying of the sample and a 60 s platinum coating. The sample was imaged using a FEI Teneo Volumescope (Hillsboro, OR, USA) field emission scanning electron microscope (SEM).

### 2.9. Transmission Electron Microscopy

An aliquot (3 μL) of the washed assembly of **5HB1** was placed gently on a 400-mesh copper grid that had been coated with a carbon film (Electron Microscopy Sciences) (Hatfield, PA, USA) and glow-discharged. The sample was absorbed for 1 min. After drying the sample, 2% uranyl acetate was used for staining (Electron Microscopy Sciences). We analyzed the assembly with a Tecnai T20 transmission electron microscope (TEM, FEI Company) (Hillsboro, OR, USA) operated at 100 KV, using a 200-μm condenser aperture, a spot size of 3, and a 70 μm objective aperture. We used a Gatan US1000 2Kx2K CCD camera to captured the resulting images (Scientific Instruments and Application) (Duluth, GA, USA).

### 2.10. Cargo Inclusion before Assembly Formation

To a microtube with water (27.1 μL) and MOPS buffer (12.5 μL, 200 mM, pH = 7.4) was added His_6_-eGFP (0.4 μL, 4.3 μg/μL) and **5HB1** (5 μL, 5 mM) followed by ZnCl_2_ (5 eq.) to promote higher-order assembly. The same procedure was followed for AONB inclusion with water (27.2 μL) and AONB (0.33 μL, 3 mM) used. The final volume of the mixture was 50 μL with final concentrations of 50 mM for MOPS buffer, 0.5 mM for **5HB1** and 2.5 mM for metal ion. The final concentration of cargo was 7 μM for His_6_-eGFP and 20 μM for AONB. The mixture was incubated at RT for 1 h followed by centrifugation at 5000× *g* for 3 min. The supernatant was removed, and the pellet was washed twice with water.

### 2.11. Cargo Inclusion after Assembly Formation

The assemblies were made following the procedure above. After washing, the assembly was resuspended in a solution of NiCl_2_ (1 mM) in water (50 μL) for 1 h. The solution was centrifuged at 5000× *g* for 3 min. The supernatant was removed and the pellet was washed twice with water. The washed pellet was then resuspended and incubated with His_6_-eGFP solution (7 μM) in water (50 μL) for 1 h. The assemblies were centrifuged at 5000× *g* for 3 min. The supernatant was removed, and the pellet was washed twice with water.

For AONB inclusion, the assemblies were resuspended and incubated with AONB solution (20 μM) in water (50 μL) for 1 h. The assemblies were centrifuged at 5000× *g* for 3 min. The supernatant was removed, and the pellet was washed twice with water.

### 2.12. Confocal Microscopy

An aliquot (3 μL) of the washed assembly of **5HB1** was placed in a 12-well chamber from ibidi GmbH and imaged on a Nikon A1R multiphoton inverted confocal microscope (Melville, NY, USA) with 40× and 60× oil objectives using a 488 nm excitation laser.

## 3. Results and Discussion

### 3.1. Peptide Design, Synthesis and Barrel Crosslinking

The de novo design of the amphiphilic peptide **L6HCH** used the design parameters of its predecessor peptide **L6HC** (Figure 1b) to make an oligomeric helical barrel [16]. The hydrophobic residue, Leu, was placed at *i* + 3 or 4 positions to form the hydrophobic face to bring the monomers together via hydrophobic interactions [28]. Individual helices were stabilized with intrahelical salt bridges between similarly placed, complimentary charged residues Glu and Lys [29,30]. The design also included minimizing repulsive interactions with charged groups at the termini of the peptide and the helix dipole [31,32,33,34]. Interhelical covalent crosslinking of the helices with disulfide bonds was also used in this design [16]. Homocysteine (HCys), a cysteine homolog, has been shown to have the optimal length for disulfide formation between helices and avoids steric hindrance between interhelical residues. Furthermore, the placement of His (Figure 1c, blue) in the center of helices was envisioned as a ligand to promote metal chelation [35], thereby leading to higher-order assembly of the helical barrels. (Figure 1c)

**Figure 2:**
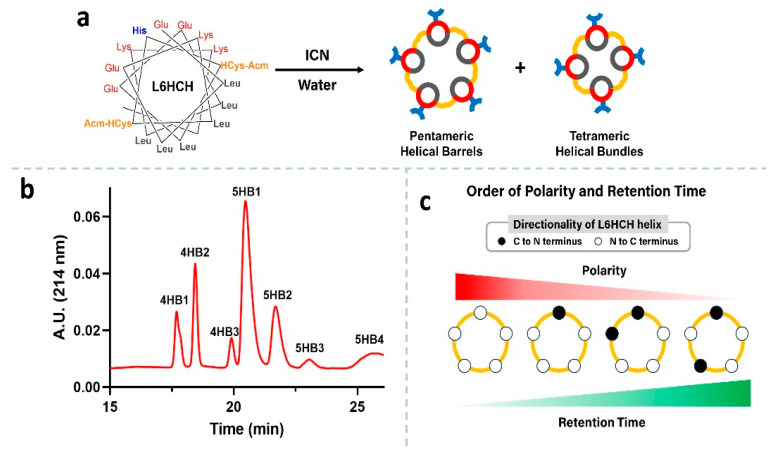
(**a**) A helical wheel representation of **L6HCH**, with the synthesis of helical bundles/barrels starting with **L6HCH** and ICN. (**b**) Chromatogram of reaction mixture peaks corresponding to pentameric helical barrels and tetrameric bundles are labeled as characterized by mass spectrometry. (**c**) Proposed order of polarity and retention time of pentameric helical bundles, from left to right, **5HB1**, **5HB2**, **5HB3** and **5HB4**.

The amphiphilic peptide, **L6HCH**, was prepared using solid-phase peptide synthesis using standard Fmoc-based conditions. After the final deprotection of the Fmoc group on resin, an acetyl group was installed at the N-terminus with acetic anhydride and DIEA. The peptide was cleaved from the resin using a TFA cocktail (95% TFA, 2.5% TIPS and 2.5% water), purified to homogeneity by RP-HPLC (>95% purity, Figures S1 and S2) and characterized by MALDI-ToF mass spectrometry (Figures S1–S3). With the monomeric peptide in hand, we removed the Acm protecting groups from the two HCys residues of **L6HCH** (0.5 mg/mL) using cyanogen iodide (100 eq.) in water for 24 h (Figure 2a) [16,21]. This process also concurrently allowed for the formation of the disulfide bonds between HCys residues on neighboring helices (Figure 2a). Following dialysis of the reaction, the mixture was separated by RP-HPLC (Figure 2b) and analyzed by MALDI-ToF mass spectrometry. The reaction was found to produce three disulfide-linked tetrameric helical bundles (**4HB1**, **4HB2** and **4HB3**) and four disulfide-linked pentameric helical barrels (**5HB1**, **5HB2**, **5HB3** and **5HB4**) (Figure 2b) as determined by mass spectrometry (Figure S5 for **5HB1**). Interestingly, one pentamer, **5HB1**, was obtained as the major product relative to the other oligomeric bundles (Figure 2b). We investigated 3 different starting concentrations of **L6HCH** (0.5, 2 and 4 mg/mL) and **5HB1** was the predominant product formed under each of these conditions, and the ratio of oligomers was also very similar in each (Figure S6).

There are four possible orientations for the helices in the disulfide-linked pentameric helical barrels. (Figure 2c) Of these, the all-parallel combination should be the most polar due to the inherent dipole moment of the individual helices that are aligned in the same direction. The resultant polarity should decrease upon introducing antiparallel helices, with the barrel containing the alternating antiparallel helices (Figure 2c—right) potentially the most nonpolar. Based on the nature of the packing material in the RP-HPLC experiment, we conjecture that **5HB1**, the helical barrel that elutes first from column, is the most polar barrel with all-parallel helices (Figure 2c—left).

### 3.2. Secondary Structure Determination and Thermal Stability

**Figure 3:**
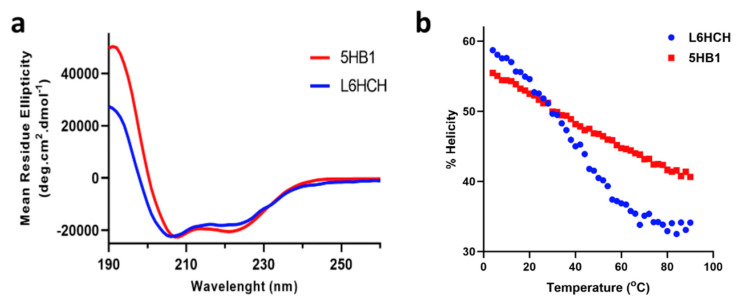
(**a**) Circular dichroism (CD) spectra of **5HB1** and **L6HCH** (25 μM) measured in phosphate buffer (20 mM, pH 7.2). (**b**) Helicity (%) plotted against temperature for **L6HCH** and **5HB1**.

The secondary structure of the peptide **L6HCH** and linked barrel **5HB1** was investigated using circular dichroism (CD) spectroscopy. Both **L6HCH** and **5HB1** (25 μM in phosphate buffer) were found to be alpha-helical in nature as suggested by the minima at 208 nm and 222 nm (Figure 3a), with similar helical contents of 53% at 22 °C. The thermal stability of **L6HCH** and **5HB1** was probed using CD with varying temperature from 4 to 90 °C. (Figure 3b) As expected, the helicity decreased with an increase in temperature in both cases; however, the extent of unfolding was more pronounced for **L6HCH**, with a sigmoidal shape denaturation curve demonstrating cooperative unfolding. On the other hand, the thermal unfolding of **5HB1** was linear, and the helical barrel still retained ~42% helicity at 90 °C. These data confirm that the helical structure of **5HB1** is somewhat more thermally stable as compared to **L6HCH** since the helices are crosslinked covalently and cannot dissociate or unfold completely.

### 3.3. Molecular Modeling

**Figure 4:**
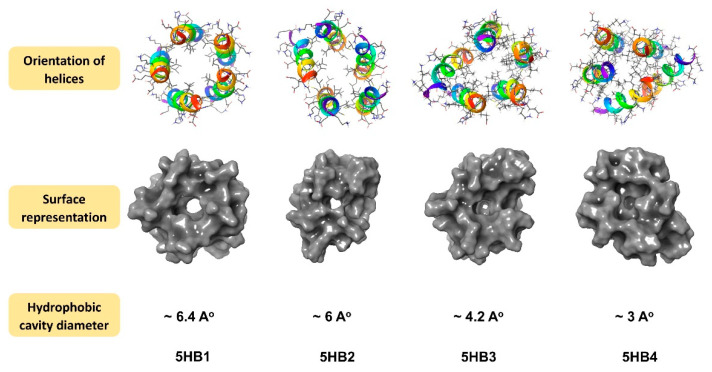
Structural analysis of the helical barrels of **5HB1**, **5HB2**, **5HB3** and **5HB4**, showing the orientation of the helices, surface representations and hydrophobic cavities (from in silico modeling using Maestro and MacroModel).

Stochastic dynamics simulations [27] using the MacroModel software package (Schrödinger release 2023-3) [23] and the AMBER94 force field [24] were performed to optimize the model structures of **5HB1** and the other proposed pentameric helical barrels, **5HB2**, **5HB3** and **5HB4**. It was found that within each optimized barrel structure, the helices orient in a tilted manner to promote ionic and hydrophobic intrahelical interactions (Figure 4). The surface of **5HB1** was found to contain a hydrophobic cavity with a diameter of ~6 Å, which is consistent with other reported structures for pentameric helical barrels [5] (Figure 4). Interestingly, the inclusion of antiparallel helices obstructed the hydrophobic cavity on one end, making these barrels slightly more oval in their overall structure and more bucket shaped as compared to the all-parallel helices in **5HB1**.

### 3.4. Inclusion of Hydrophobic Dyes

**Figure 5:**
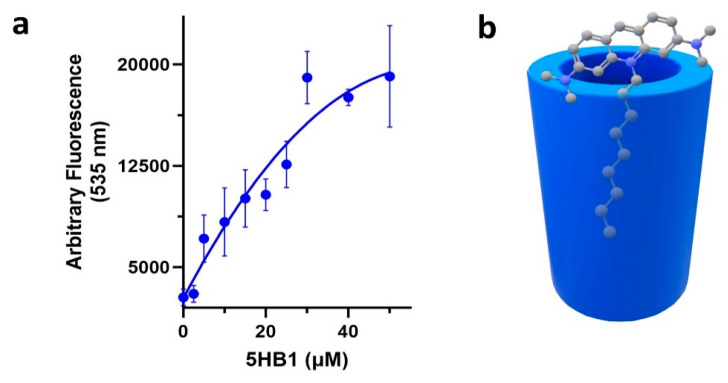
(**a**) Fluorescence intensity measurements of AONB (1 μM) at 535 nm as a function of the **5HB1** concentration. (**b**) Schematic representation of AONB bound within the hydrophobic cavity of **5HB1**.

Helical barrels have been shown to contain a hydrophobic cavity that can encapsulate hydrophobic molecules [5,11,14,16,36]. To validate this for our design (Figure 5), we chose a dye that responds to a hydrophobic environment and contains a hydrophobic alkyl tail that may bind into the central hydrophobic cavity of **5HB1**, acridine orange nonyl bromide (AONB) [16,18]. In this way, the hydrophobic tail binding within the cavity may act to shield the acridine moiety from aqueous solution at the top of the barrel, resulting in an increase in fluorescence [16,18]. An aqueous solution of AONB (1 μM) was treated with varying concentrations of **5HB1** (1–50 μM) and the fluorescence intensity was monitored (Figure 5a). As the concentration of **5HB1** was increased, we observed a concomitant increase in emission intensity of AONB. It is likely that in aqueous solution the hydrophobic tale of AONB inserts into the hydrophobic cavity of **5HB1**, thus bringing the acridine moiety close to the rim of the barrel and the non-polar environment from the hydrophobic cavity composed of Leu sidechains (Figure 5b).

### 3.5. Metal-Promoted Higher-Order Assembly of 5HB1

**Figure 6:**
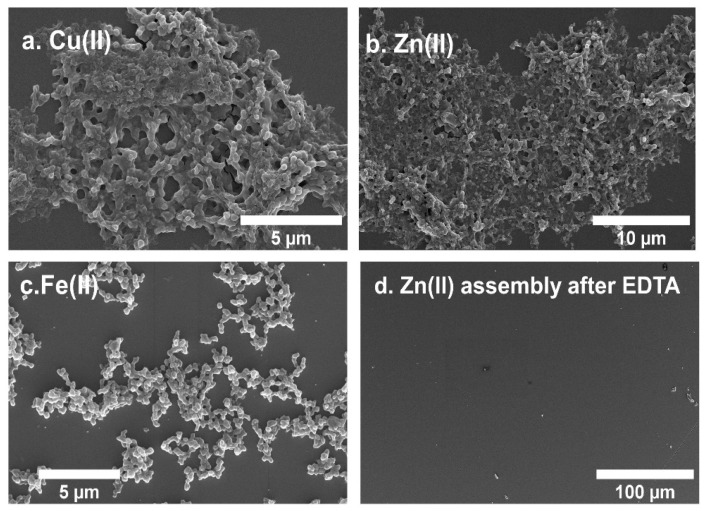
SEM images of the metal-promoted assembly of 5HB1 (0.5 mM) in MOPS buffer (40 mM, pH 7.4) with (**a**) Cu(II), (**b**) Zn(II), (**c**) Fe(II) and (**d**) addition of EDTA (100 eq) to the Zn-promoted assembly.

With **5HB1** as a building block containing five His residues for metal chelation (one from the middle of each helix), we next evaluated the metal-dependent assembly of **5HB1** into higher-order structures. We evaluated a series of bivalent metal ions (Zn(II), Co(II), Ni(II), Fe(II) and Cu II)) starting with **5HB1** (0.5 mM) and two equivalents of each metal using MOPS buffer (50 mM, pH 7.4) at RT (Figure 6). The clear solution of **5HB1** turned cloudy immediately after addition of Zn(II) and Cu(II) and after 30 min with Fe(II). Addition of Co(II) and Ni(II) did not produce a precipitate after 24 h. After a 1 h incubation, the solutions were centrifuged and the pellets were washed prior to SEM imaging (Figure 6). The SEM images of the Cu(II)- and Zn(II)-promoted assemblies of **5HB1** displayed a morphology that is consistent with a three-dimensional (3D) matrix composed of interconnected spheres. These structures extended over hundreds of microns and contained inner cavities in the low micron to high nm range. TEM of the Zn(II) assembly also confirmed the morphology observed by SEM (Figure S7). The Fe(II) assembly also was composed of connected spheres (Figure 6a–c), but the structures were much more dispersed. We also performed additional assembly experiments varying the equivalents of metal ion (Figures S8–S10), temperature and peptide concentration, but these conditions did not significantly change the morphology of the assemblies obtained.

To verify that the assembly was indeed metal promoted, we treated the Zn(II) assembly with ethylenediamine tetraacetate (EDTA), a strong metal chelator, which should chelate the metal ions. After the addition of EDTA to the 3D matrix, we observed an immediate dissolution of the assembly both visually and by SEM (Figure 6d), demonstrating the crucial involvement of metal ions in the formation of the assembly. It is also worth noting that **5HB1** did not assemble with Zn(II) at pH 6.0 in the MES buffer, and the assembly was rapidly degraded under acidic conditions (citrate buffer, pH 3.0). Since the pKa of the imidazole sidechain of His is ~6, these data demonstrate the importance of the imidazole ligands, and potentially other nearby carboxylate ligands [37], within **5HB1** on the metal ion-promoted higher-order assembly.

### 3.6. 3D-Matrix Cargo Loading

**Figure 7:**
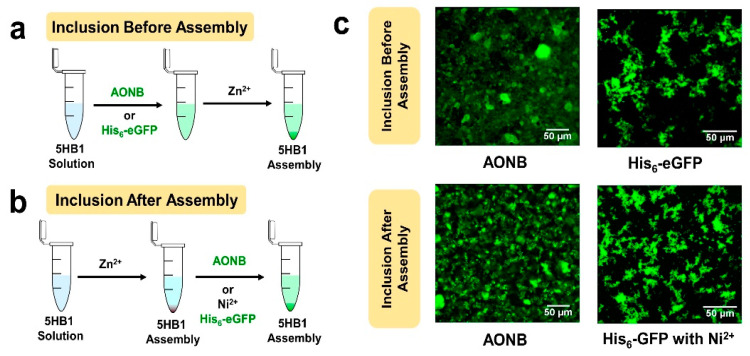
Methods of cargo loading of a hydrophobic dye (AONB) or His-tagged fluorescent protein within the 3D matrix of **5HB1** both (**a**) before/during assembly and (**b**) after assembly. (**c**) Confocal microscopy images of the materials obtained from the before and after methods with the two fluorophores.

Since the 3D matrix formed with **5HB1** and metal ions contains a cavity within the helical barrel building block, and also has the potential for unsatisfied ligands for metal ions within and on the surface of the matrix, we have explored this material for loading of a hydrophobic dye and a His-tagged protein as cargo. We first investigated the incorporation of AONB into the 3D matrix of **5HB1**, both before and after the metal-promoted assembly (Figure 7). **5HB1** that was pre-treated with AONB followed by treatment with Zn(II) forms fluorescent assemblies (Figure 7c—top left) with the same morphology found above (Figure S11). Similar results were observed when the 3D matrix was treated with AONB after assembly (Figure 7c—bottom left). (Figure 7a and Figure S12) These data indicate that the helical barrel pores can be decorated with the hydrophobic dye both before and after assembly while not affecting the overall morphology of the 3D matrix.

Similarly, unsaturated coordination sites for the metal ions in the higher order structure of **5HB1** could be used to incorporate His-tagged proteins both during and after assembly (Figure 7), with the His-tag serving as a ligand to bring the protein within the matrix in a metal-dependent fashion. For that purpose, we chose His-tagged eGFP to allow for easy visualization of binding with fluorescent confocal microscopy. His_6_-eGFP (7 μM) was added to the assembly mixture of **5HB1** and Zn(II) (Figure 7a), and again facile assembly of fluorescent material was observed (Figure 7c—top right) without disrupting the morphology of the 3D matrix (Figure S13). To incorporate His-tagged eGFP after the assembly has formed (Figure 7b), the 3D mesh was first incubated with Ni(II) to provide metal-coordinated ligands within the matrix, and, after removing the excess Ni(II), the assembly was incubated with His-tagged eGFP. This process introduced the fluorescent protein within the 3D matrix (Figure 7d—bottom right) without disrupting the morphology of the assembly (Figure S14). This strategy of using unsatisfied metal/ligands within the biomaterial to bring His-tagged proteins within the matrix is very powerful and may allow for the inclusion of multiple proteins into the same assembly [38,39].

## 4. Conclusions

Herein, we have described the development of a pentameric, helical barrel building block with augmented thermal stability due to disulfide staples and His residues available for metal-promoted assembly. Molecular modeling studies indicated the presence of a hydrophobic cavity roughly 6 Å in diameter within one pentamer, **5HB1**, that is lined with isobutyl groups from Leu residues along the interior face of the helical peptides (Figure 2a and Figure 4—left). The binding of a hydrophobic dye to the helical barrel was established, consistent with the predictions of a hydrophobic cavity from the molecular modeling studies. Further, we have demonstrated that metal ions promote the higher order assembly of the barrel building block, **5HB1**, to a 3D matrix structure over hundreds of microns in size with nano- to micro-sized clefts. Finally, we developed two routes for cargo incorporation (dyes and proteins) within the matrix using either the hydrophobic barrel core or unsatisfied ligands within the structure, both before and after assembly. This work sets the stage to use helical barrel matrices as an important scaffold for biomaterial applications using a mesh morphology.

## Data Availability

Available from the corresponding authors.

## Supplementary Materials

The following supporting information can be downloaded at https://www.mdpi.com/article/10.3390/nano13192645/s1. Figures S1–S5: HPLC traces of crude and pure **L6HCH** and pure **5HB1** and mass spectra. Figure S6: Distribution of crosslinked products at varied monomeric peptide concentrations. Figure S7: TEM images of the **5HB1** (0.5 mM) assembly with Zn(II) (2 eq.). Figures S8–S10: SEM images of the **5HB1** (0.5 mM) assembly with metal ions (5 eq.). Figures S11 and S12: SEM images of the **5HB1** (0.5 mM) assembly with Zn(II) and AONB added before and after assembly. Figures S13 and S14: SEM images of the **5HB1** (0.5 mM) assembly with Zn(II) and His-tagged eGFP added during and after assembly.
