# Supplementary material for: Metal-Promoted Higher-Order Assembly of Disulfide-Stapled Helical Barrels
Source: Nanomaterials (Basel). 2023 Sep 26;13(19):2645. doi: 10.3390/nano13192645 (PMC10574645; doi:10.3390/nano13192645)
Supplement: Supplementary file 1 [file nanomaterials-13-02645-s001.zip › nanomaterials-2618298-supplementary.pdf]

Metal-Promoted Higher-Order Assembly of Disulfide-Stapled Helical Barrels

Ashutosh Agrahari, Mark Lipton*, Jean Chmielewski*

560 Oval Drive, Department of Chemistry, Purdue University, Indiana, USA

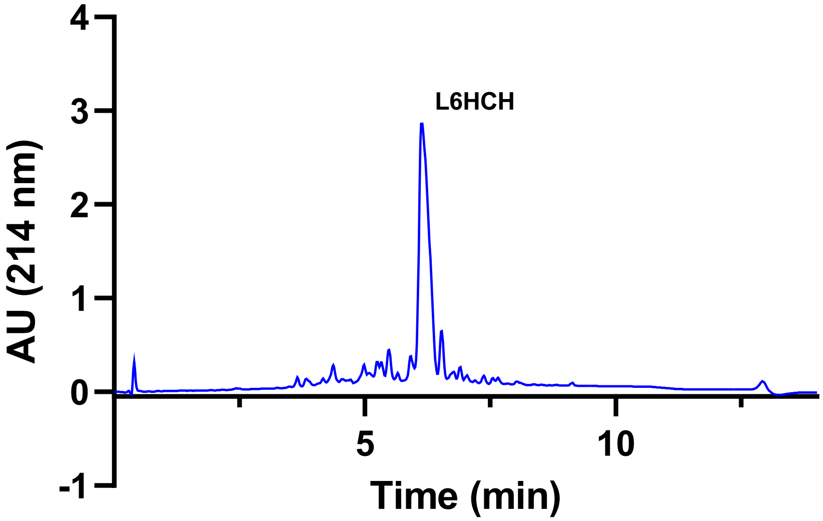

Figure S1. RH HPLC of crude of **L6HCH** synthesis with a solvent gradient of 10-80% acetonitrile/water (0.1% TFA) over 10 min.

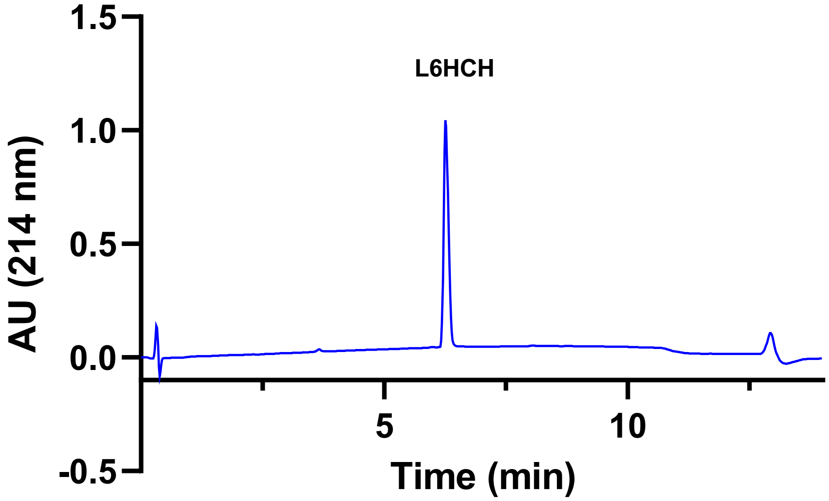

Figure S2. RP HPLC trace of purified **L6HCH** with a solvent gradient of 10-80% acetonitrile/water (0.1% TFA) over 10 min.

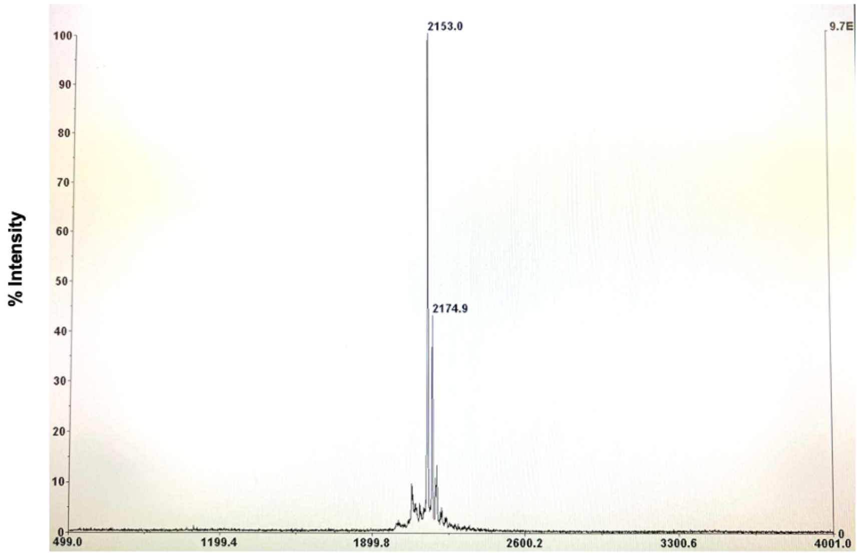

Figure S3. MALDI mass spectrum of purified **L6HCH**.

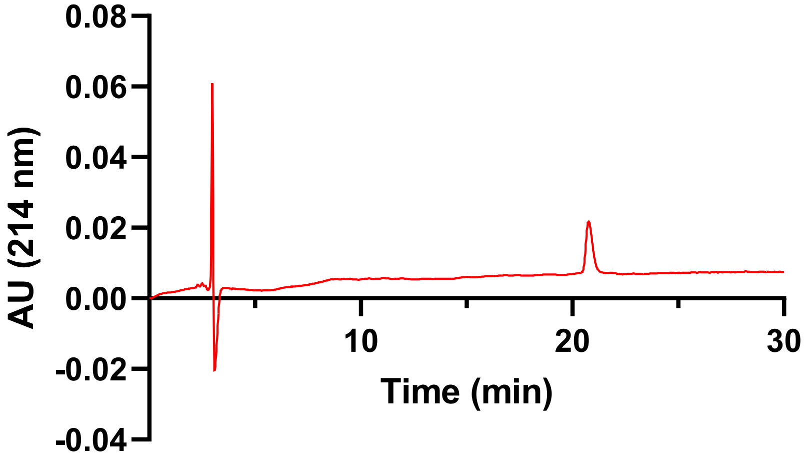

Figure S4. RP HPLC trace of purified of **5HB1** with a solvent gradient of 10-80% acetonitrile/water (0.1% TFA) over 30 min.

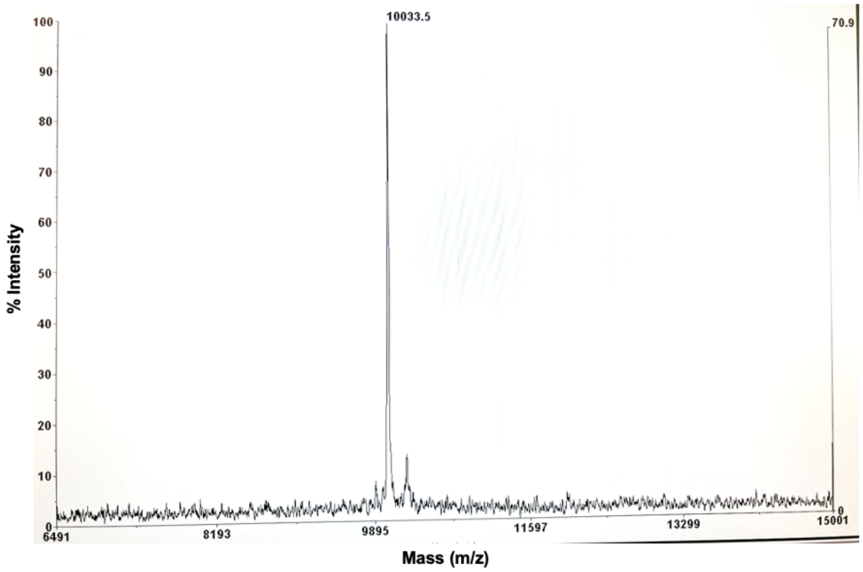

Figure S5. MALDI mass spectrum of purified **5HB1**.

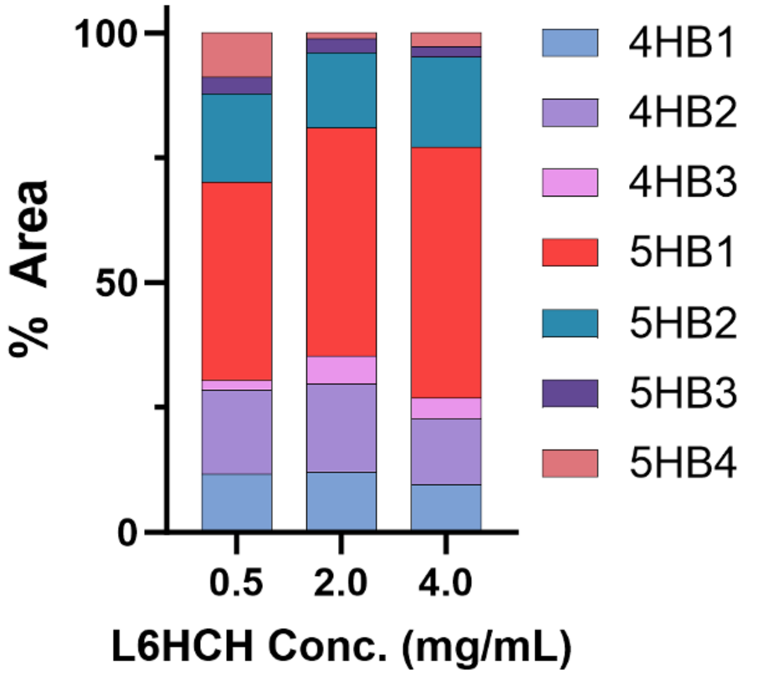

Figure S6. Distribution of helical bundles and barrels in the crosslinking reaction of **L6HCH** at 3 different monomer concentrations.

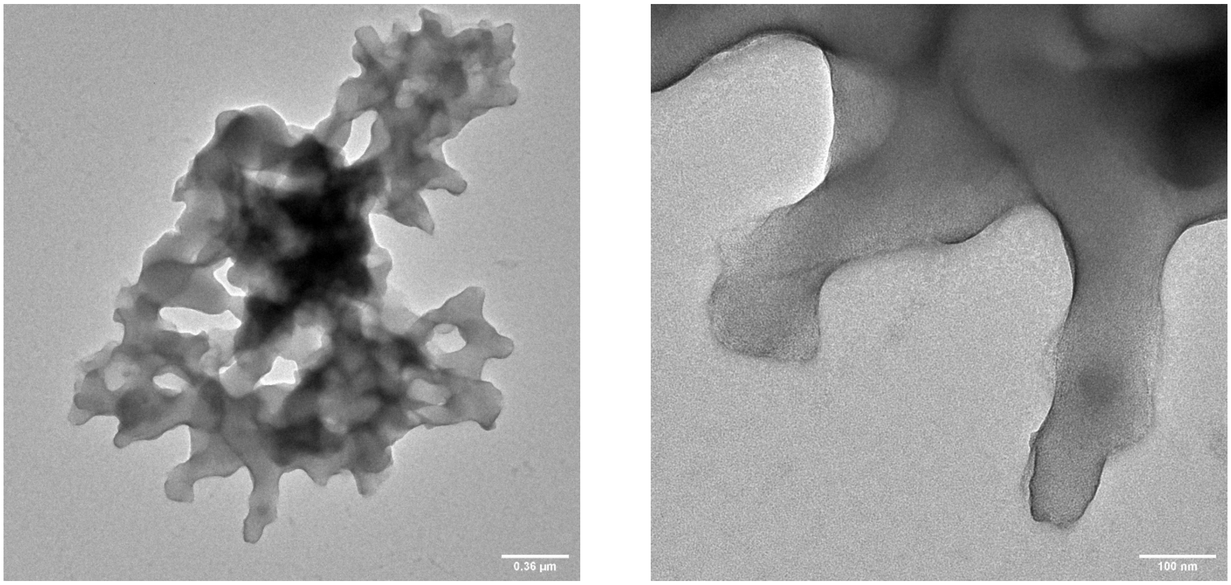

Figure S7. TEM images of **5HB1** (0.5 mM) assembly with Zn(II) 2 eq. in MOPS buffer after 1 h incubation.

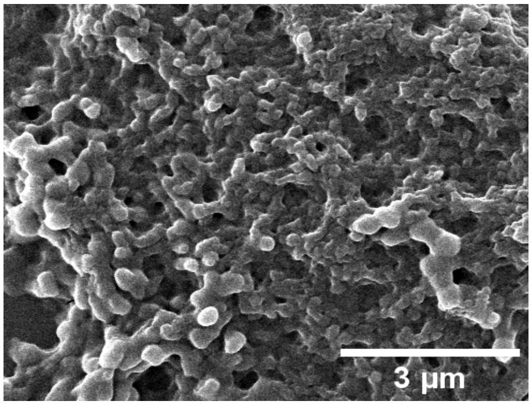

Figure S8. SEM image of **5HB1** (0.5 mM) assembly with Cu(II) (5 eq.) in MOPS buffer after 1 h incubation.

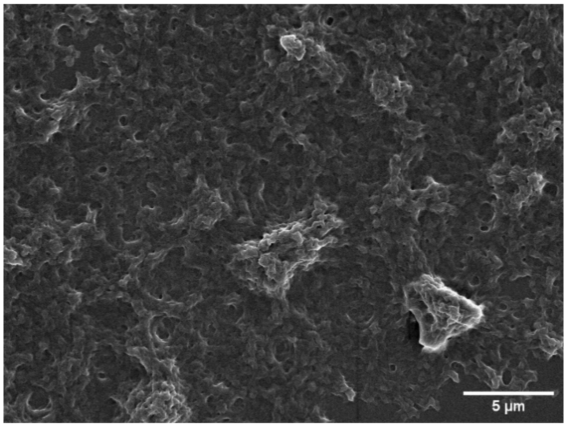

Figure S9. SEM image of **5HB1** (0.5 mM) assembly with Zn(II) (5 eq.) in MOPS buffer after 1 h incubation.

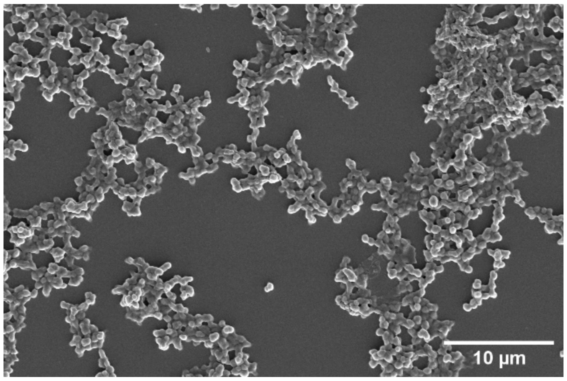

Figure S10. SEM image of **5HB1** (0.5 mM) assembly with Fe(II) (5 eq.) in MOPS buffer after 1 h incubation.

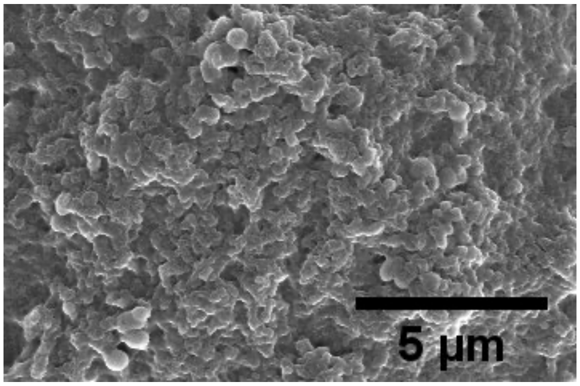

Figure S11. SEM image of **5HB1** (0.5 mM) assembly with AONB (20 μM), Zn(II) (5 eq.) in MOPS buffer after 1 h incubation.

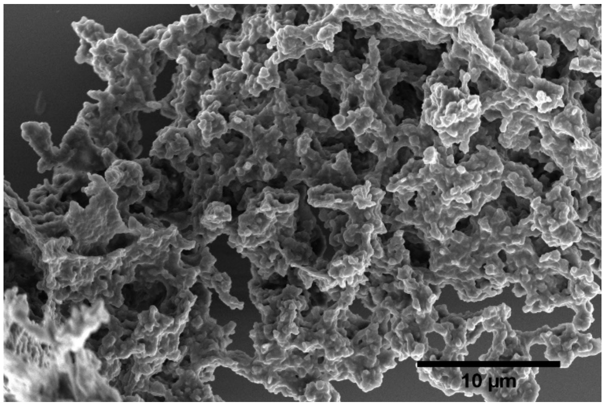

Figure S12. SEM image of **5HB1** (0.5 mM) assembly with Zn(II) (5 eq.) in MOPS buffer after 1 h incubation, post treatment with AONB (20 μM) .

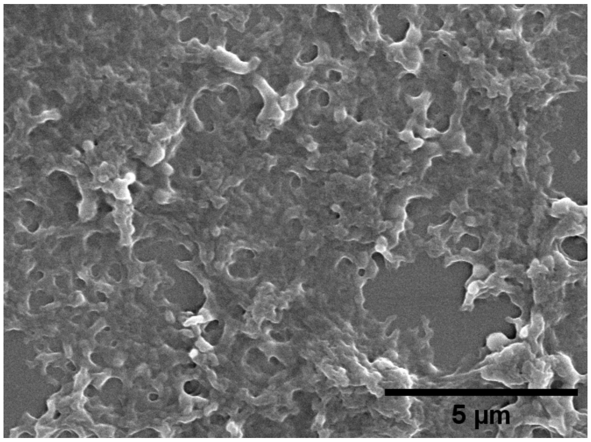

Figure S13. SEM image of **5HB1** (0.5 mM) assembly with His_6-eGFP (7 μM) and Zn(II) (5 eq.) in MOPS buffer after 1 h incubation.

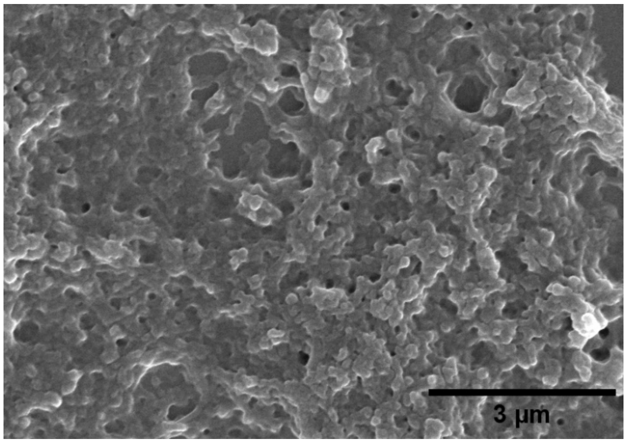

Figure S14. SEM image of **5HB1** (0.5 mM) assembly with Zn(II) (2 eq.) in MOPS buffer after 1 h incubation, post treatment with Ni(II) (2 eq.) and His_6-eGFP (7 μM).
